# Determining the microenvironment and protonation state of quercetin encapsulated in pillar[5]arene-based supramolecular nanocarriers

**DOI:** 10.1039/d6ra02504h

**Published:** 2026-05-26

**Authors:** Marco Milone, Martina Mazzaferro, Salvatore Patanè, Anna Notti, Ilenia Pisagatti, Giuseppe Gattuso, Norberto Micali, Valentina Villari

**Affiliations:** a Dipartimento di Scienze Chimiche, Biologiche, Farmaceutiche ed Ambientali, Università degli Studi di Messina Viale F. Stagno d'Alcontres 31 98166 Messina Italy; b Dipartimento di Scienze Matematiche e Informatiche, Scienze Fisiche e Scienze della Terra, Università degli Studi di Messina Viale F. Stagno d'Alcontres 31 98166 Messina Italy; c CNR-IPCF Consiglio Nazionale delle Ricerche, Istituto per i Processi Chimico-Fisici Viale F. Stagno d'Alcontres 37 98158 Messina Italy valentina.villari@cnr.it

## Abstract

This study reports the optical properties of quercetin encapsulated in self-assembled nanoparticles made from a dicarboxyl-bis-pillar[5]arene/CTAB supramolecular complex, and correlates these properties with the nanoparticle architecture and microenvironment. Experimental evidence indicates that quercetin, in both its neutral and mono-anionic form, is mostly located within the nanoparticle, trapped between the pillararenes. In contrast, only a small fraction of the mono-anionic form is found on the nanoparticle surface. Quercetin is stabilized through hydrogen bonding and hydrophobic interactions, which play a crucial role in protecting the flavonol from oxidation and degradation. This stabilization explains our recent findings on the efficacy of these nanoparticles in delivering and facilitating the cellular internalization of quercetin.

## Abstract 2

Pillararene host–guest complexes organize into robust nanocarriers able to control the location and speciation of encapsulated quercetin.

## Graphical abstract

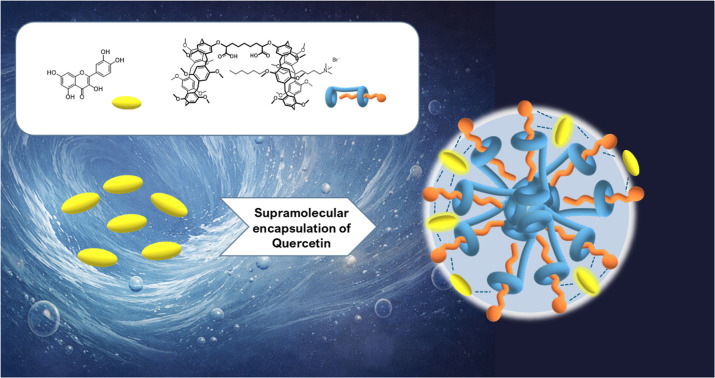

## Introduction

Nanostructured systems are among the most powerful tools in modern materials science, enhancing sustainability, sensing capabilities, optoelectronic properties, and biomedical applications. In pharmaceuticals, one key objective of studying nanostructures is to improve the bioavailability, stability, and efficacy of poorly water-soluble drugs for drug delivery applications.^1,2^ Among the various nanocarrier platforms developed in recent years, special attention has been given to systems capable of combining high drug loading capacity with low toxicity and stable formulation. In this context, supramolecular assembly based on macrocyclic hosts has emerged as a highly versatile strategy for designing responsive and tunable delivery platforms.^3^ Over the past decade, pillar[*n*]arenes,^4–6^ have established themselves as versatile supramolecular building blocks for constructing a wide range of delivery systems, including micelles, vesicles, solid lipid nanoparticles, and polymeric nanoparticles.^7–11^ Pillar[*n*]arenes are a class of paracyclophanes composed of methylene-bridged hydroquinone rings (*n* = 5, 6), characterized by a rigid, highly symmetrical architecture and an electron-rich hydrophobic cavity. They allow for easy rim functionalization and have a remarkable propensity for host–guest inclusion of linear alkyl chains. These features make them particularly well suited for constructing supramolecular amphiphiles and self-assembled nanostructures for delivering a variety of drugs and bioactive molecules. Despite the aromatic and intrinsically hydrophobic nature of the pillararene scaffold, water-soluble derivatives can be readily obtained through rim functionalization with ionic or hydrophilic groups, yielding biocompatible macrocycles with low cytotoxicity and excellent colloidal stability. These derivatives enable the development of supramolecular nanoparticles capable of drug encapsulation *via* a combination of host–guest inclusion, hydrophobic interactions, and electrostatic assembly. Importantly, the modular nature of pillararene chemistry allows fine control over nanoparticle size, surface charge, internal microenvironment, and guest binding strength, which are all key parameters that influence formulation stability and biological performance. In our previous work,^12^ we reported the preparation of water-dispersible supramolecular nanoparticles based on the host–guest complexation between a dicarboxyl-bis-pillar[5]arene derivative (H) and the surfactant cetyltrimethylammonium bromide (CTAB). The supramolecular CTAB/H complex is formed through hydrophobic inclusion of the CTAB alkyl chain into the pillararene cavity, resulting in a stable aqueous solution. Dynamic light scattering (DLS) and electrophoretic measurements revealed that this complex spontaneously forms monodisperse nanoparticles with a hydrodynamic radius of approximately 40 nm, a positive surface charge, and long-term colloidal stability. These nanoparticles have been shown to efficiently encapsulate the poorly water-soluble flavonoid quercetin (Q), while maintaining low cytotoxicity and promoting cellular internalization.

**Scheme 1:**
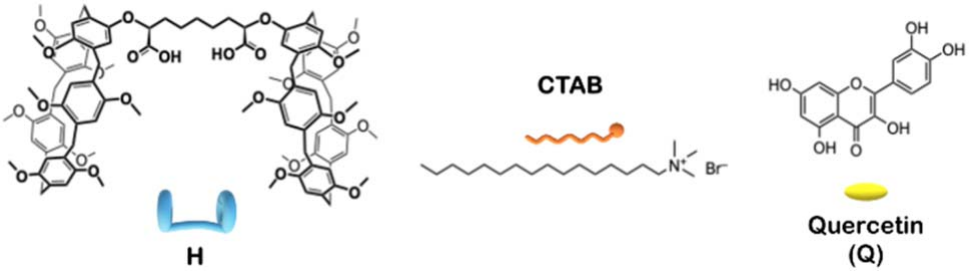
Structures of the dicarboxyl-bis-pillar[5]arene derivative (H), quercetin (Q) and cetyltrimethylammonium bromide (CTAB).

Quercetin^13^ is a flavonol well-known for its antioxidant, anti-inflammatory, cardioprotective, neuroprotective, and anticancer properties. However, its pharmaceutical applications are severely limited by extremely low water solubility, poor chemical stability, rapid degradation in physiological media, and low oral bioavailability.^14,15^ Building on our previous findings, the present work aims to provide a structural and spectroscopic characterization of the CTAB/dicarboxyl-bis-pillar[5]arene supramolecular nanoparticles (see Scheme 1 for the molecular structures) and to elucidate the molecular interactions governing quercetin encapsulation and stabilization. In particular, we have investigated the absorption and emission properties of free, CTAB-associated, and nanoparticle-loaded quercetin, correlating the spectroscopic response with nanoparticle architecture and microenvironment. Fluorescence spectroscopy, in particular, is very sensitive to changes in the molecular environment of quercetin at the concentration investigated in the present work. Furthermore, the characterization techniques have been employed to hypothesize the localization of quercetin within the supramolecular carrier and to evaluate the stability of the encapsulated flavonoid.

## Materials and methods

### Preparation of CTAB/H nanoparticles

Bis-pillar[5]arene H was prepared according to a literature procedure.^16^ CTAB/H solutions were prepared by adding 200 µL of an H stock solution (20 mM in chloroform) to 10 mL of an aqueous solution of CTAB (0.9 mM). The solvent evaporation method was then followed, using probe sonication for 25 min to minimize the chloroform content. For comparison, the same method was used to prepare CTAB solution without H. In the final aqueous solutions, the concentration of the residual chloroform traces is well below the NMR detection limit (typically 1–10 µM).^17^ According to ref. 12, an aliquot (0.8 mL) of these solutions was subjected to solid–liquid extraction experiments with 1 mg of quercetin, stirred and centrifuged to recover the supernatant.

Finally, the obtained solutions were diluted in water at a 1 : 10 ratio.

A free quercetin aqueous solution was prepared by dissolving a weighted amount of solid (1 mg) in water (4 mL), stirring for 4 h at room temperature, then centrifuging at 5000 rpm for 15 min to eliminate the micrometric and millimetric undissolved clusters, and finally collecting the supernatant.

The pH of all solutions was found to be 6.9 ± 0.1 and the prepared solutions were used within a day from preparation.

### Instruments and data analysis

Absorption spectra were measured using an Avantes Avaspec spectrometer with a resolution of 0.8 nm and deuterium–halogen lamp (Avalight) as the source.

Excitation and steady-state fluorescence spectra were collected by a Jasco FP-8350ST fluorimeter. By inserting a polarizer along the incident path to select the vertically polarized light, and an analyser (followed by a depolarizer) along the emitted path, the vertically (*I*_VV_) and horizontally (*I*_VH_) polarized contributions of the emission were measured. The fluorescence anisotropy was estimated as: *r* = (*I*_VV_ − *I*_VH_)/(*I*_VV_ + 2*I*_VH_).

The Raman contribution from the solvent at 456 nm, appearing in the excitation spectra with emission at 540 nm, was subtracted by considering the excitation spectra collected from a water sample.

Time-resolved fluorescence measurements were carried out using a homemade setup exploiting a polarized femtosecond Ti:Sa laser (Mai Tai - Spectra Physics) with a duplication crystal as excitation source, a monochromator (Oriel), and a microchannel-plate photomultiplier (Hamamatsu), operating in single-photon counting mode, to collect the emitted fluorescence. EG&G electronic devices were used to manage pre-amplification, constant fraction discrimination, Time-to-Amplitude Conversion (TAC), and acquisition of time-resolved fluorescence curves by means of the time correlation single photon counting method. These curves were fitted through a nonlinear least-squares iterative reconvolution according to the multiexponential law 
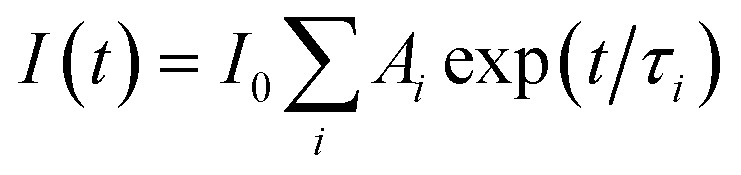
, where *I*_0_ is the fluorescence intensity at time zero, and *A*_*i*_ and *τ*_*i*_ are the relative intensity and lifetime of the *i*-th component, respectively, with 
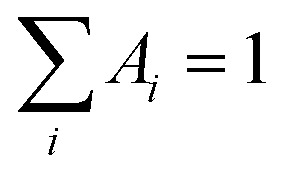
. The instrumental resolution after this reconvolution procedure is on the order of tens of picoseconds. The average lifetime was calculated as: 
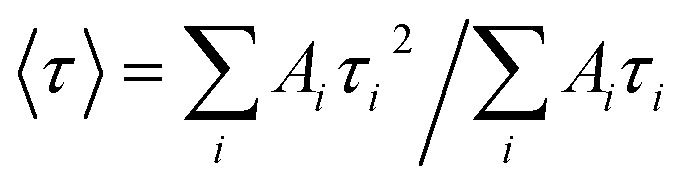
. More details can be found elsewhere.^18^ Unfortunately, slight photobleaching of quercetin under the laser beam precluded the possibility of extracting information on the time-resolved fluorescence anisotropy.

All absorption and emission measurements were performed using a quartz cuvette with a 3 mm of optical path. The absorbance values were scaled to a 1 cm-path for clarity.

The particle zeta-potential, *ζ*, was measured using a Brookhaven Zeta PALS instrument which employs the phase analysis light scattering method (see ref. 19 and 20 for details).

Atomic force microscopy (AFM) measurements were performed using an NT-MDT SMENA instrument operating in tapping mode. A silicon cantilever specifically designed for tapping mode was employed, with a resonance frequency in the 200–300 kHz range and an apical radius of approximately 5 nm. Samples were prepared by depositing a single drop of solution onto a polished silicon substrate, followed by drying under ultra-high vacuum (UHV) conditions for 30 minutes. For photoluminescence (PL) measurements, samples were prepared using an analogous procedure, with the solution deposited onto a quartz substrate to avoid unwanted signals from the substrate. The PL measurements were performed using a confocal optical setup. Excitation was provided by a 473 nm solid-state laser with an output power of 30 mW; however, to minimize photobleaching effects, the optical power reaching the sample was reduced to below 200 µW using a neutral filter. The emitted photoluminescence was collected using a 100× Mitutoyo long working distance objective (NA = 0.75), coupled to a SOL MRS750 spectrometer and detected with an Andor SOLIS IDUS CCD camera. The optical setup included a set of filters designed to efficiently suppress the excitation laser signal before it reached the spectrometer, thereby significantly improving the overall dynamic range of the measurement, similarly to what is commonly implemented in Raman spectroscopy. The resulting intensity map corresponds to the spectral integral of the emission signal in the 540–650 nm range, roughly matching the emission spectrum width of the material.^21^ The PL image consists of 128 × 128 points, with each point acquired over an integration time of 0.5 seconds.

## Results and discussion

### Spectroscopic features

**Fig. 1:**
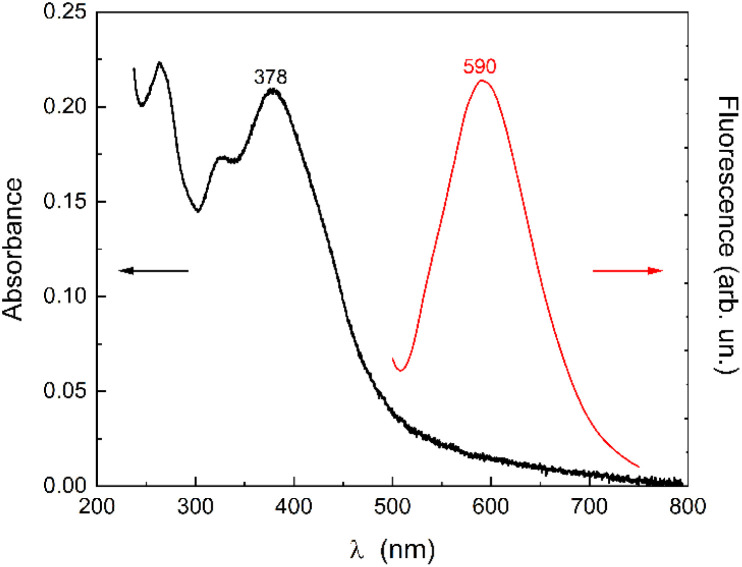
Absorption and emission (*λ*_ex_ = 370 nm) spectrum of the quercetin aqueous solution (supernatant).

The supernatant quercetin aqueous solution displays a broad absorption band centred at 378 nm (ascribed to the band I of the quercetin cinnamoyl system) and a band at 260 nm (band II – benzoyl system), along with a noticeable scattering contribution (Fig. 1). The scattering likely originates from residual suspended submicrometric clusters of undissolved quercetin. The broadening of the band I can be attributed to the coexistence of neutral (typically in the range 365–380 nm, depending on the solvent) and anionic forms (above 390 nm) of quercetin (being quercetin p*K*_a1_ ≈ 6.5), as well as degradation products from oxidation, consistent with the presence of the small band at 320 nm.^22–24^ Quercetin is known to be unstable in aqueous solutions, especially at neutral and basic pH.^25,26^ The auto-oxidation of quercetin leads to the gradual disappearance of band I over time (Fig. S1a-SI).

**Fig. 2:**
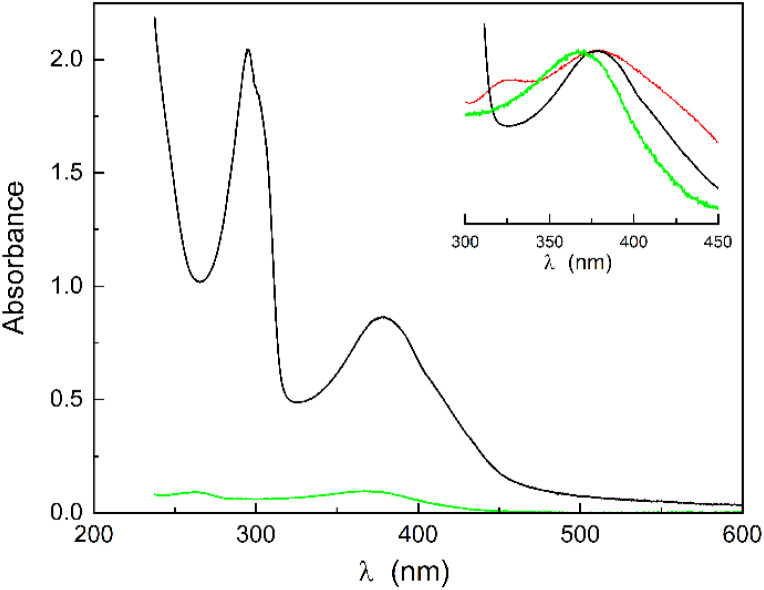
Absorption spectrum of CTAB/H/Q (black) and CTAB/Q (green), the band at about 300 nm of CTAB/H/Q belonging to H. The inset displays the peak-normalized absorption spectra compared with that of Q (red).

When quercetin is solubilized in the presence of CTAB/H nanoparticles, no shift of the absorption band centred at 378 nm is observed, but the width of this band is significantly narrower than that of the free quercetin (Fig. 2 and inset). This indicates that the interaction with the CTAB/H nanoparticles prevents contact with water, reducing the presence of multi-anionic forms and intermediate degradation products.

It is important to note that this interaction differs significantly from that of quercetin with CTAB alone in water. The CTAB concentration is below the critical micelle concentration (cmc), resulting in a very low amount of solubilized quercetin in the absence of H. However, there is a noticeable blue-shift to 370 nm in the absorption band of quercetin (not present in the CTAB/H solution), a strong reduction in the contribution of all anionic forms, and the disappearance of the band at 320 nm.

**Fig. 3:**
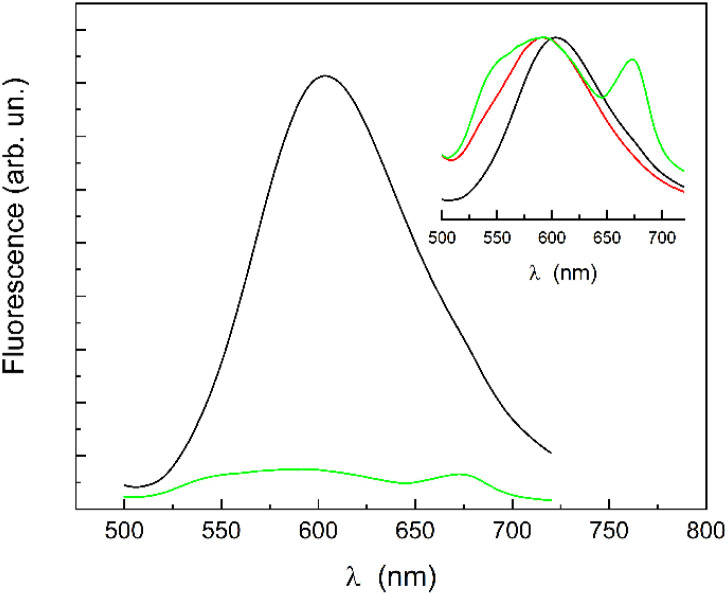
Emission spectra (*λ*_ex_ = 370 nm) of CTAB/H/Q (black) and CTAB/Q (green). The inset displays the peak-normalized emission spectra, compared with that of quercetin (red) reported in Fig. 1.

**Fig. 4:**
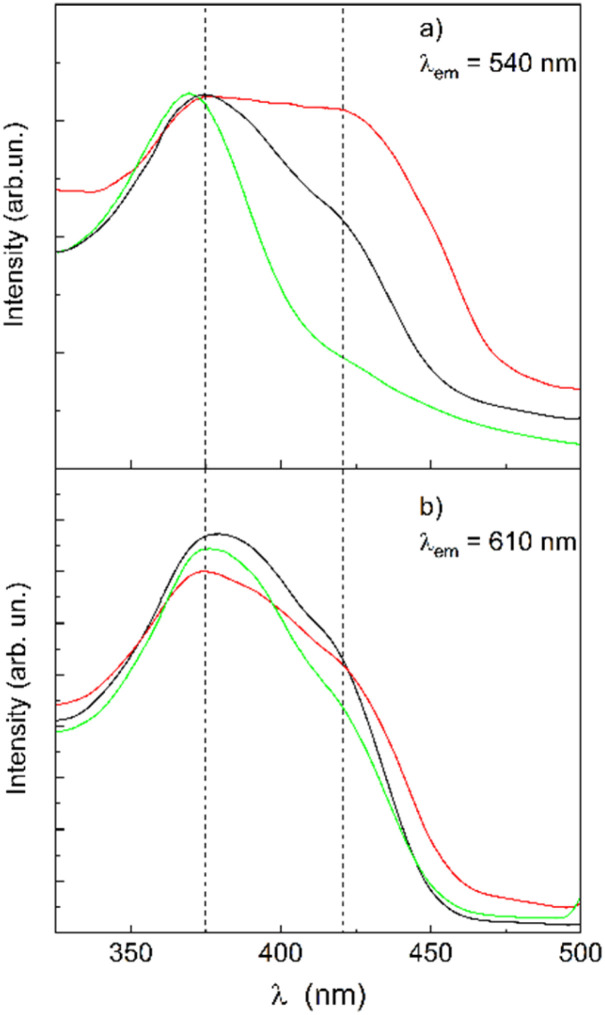
Peak-normalized excitation spectra of CTAB/H/Q (black), CTAB/Q (green) and Q (red) at two emission wavelengths: *λ*_em_ = 540 nm (a) and *λ*_em_ = 610 nm (b). The dashed lines allow for an easier comparison among the spectra at the two main absorption contributions.

This suggests an interaction between quercetin and CTAB in the absence of H. Determining the nature of this interaction is complex, but key insights can be gained from the emission spectra. The emission band of quercetin in water (excitation at 370 nm) appears at 590 nm, with a barely visible shoulder at 540 nm (Fig. 1). In contrast, in the presence of CTAB, the two quercetin bands exhibit nearly equal amplitude (Fig. 3 and inset). The contribution at 540 nm in the presence of CTAB is essentially due to the emission of the neutral form, as confirmed by the excitation spectrum (Fig. 4a), in which the band at 370 nm is predominantly excited.

There is also a peak at about 670 nm, previously observed in hydrated quercetin powder,^27^ which can be attributed to the presence of nanometric clusters of quercetin stabilized by CTAB. Indeed, as evidenced in Fig. 1, the scattering contribution from the quercetin aqueous solution (supernatant) showed that submicrometric undissolved clusters (which are not eliminated by centrifugation) coexist with dissolved molecules. It is plausible that CTAB interacts also with them, improving their stabilization in water. In the presence of CTAB/H, the amount of solubilized quercetin increases significantly and the contribution from the nanoclusters, if they persist, becomes negligible in the emission spectra of CTAB/H/Q.

The two bands at 540 and 590 nm are due to the emission of the excited state tautomers (phototautomers) of the neutral form of quercetin originated from the intramolecular proton transfer (ESIPT), as already described in several studies (see, for example, ref. 28–31). The interaction with CTAB appears to affect the radiative pathway of at least one of the tautomers. Notably, the emission at 540 nm was observed as the only band when CTAB is in the micellar form.^32,33^

Here, well below the CTAB cmc, the emission at 590 nm is still present. The preponderance of the neutral form can be explained by the existence of a less polar environment determined by hydrophobic interaction with the CTAB tails and submicromolar traces of residual chloroform, which can act as a solvation shell for the CTAB-associated quercetin. Consequently, the flavonol is not protected from water contact, leading to degradation over time (Fig. S1b-SI).

The emission at 540 nm disappears when quercetin is solubilized in the presence of CTAB/H and the main band at 590 nm undergoes a red shift to 603 nm (as evident in the inset of Fig. 3). In this case the steady-state fluorescence anisotropy of quercetin (*λ*_ex_ = 370 nm, *λ*_em_ = 610 nm) is 0.35, which is higher than the value obtained with CTAB alone (0.2). This high value suggests that most quercetin molecules are trapped within the CTAB/H nanoparticles, limiting their direct interaction with CTAB. Indeed, being CTAB included inside the cavity of H,^12^ it is less available for binding, and quercetin most likely interacts with H, which has many available sites for hydrogen bonding and hydrophobic (π–π) interactions with the flavonol.

The excitation spectra in Fig. 4 indicate that both neutral and anionic forms of quercetin are present in the CTAB/H nanoparticles. Therefore, hydrogen-bonded neutral quercetin or the mono-anionic species can be identified as the primary contributors to the emission at 603 nm (and the absorption band centred at 378 nm). The CTAB/H nanoparticles provide both electronic stabilization through specific H-bonding (for example, with the pillararene carboxyl groups) and rigidity, which, by restricting quercetin intramolecular rotations, ‘locks’ the guest into a stable conformation. Indeed, the carboxylic groups of H are not necessarily exposed to the aqueous phase and are located in a relatively hydrophobic environment within the nanoparticles. Such an arrangement may limit their effective ionization, since the hydrophobic environment favours the neutral carboxyl form over the deprotonated one. In addition, traces of the sub-micromolar residual chloroform may remain embedded in such a region, further hindering carboxyl deprotonation, as well as stabilizing the neutral form of quercetin, as suggested by the differences observed in the excitation spectra for free and loaded quercetin in Fig. 4. Consequently, within the confined and heterogeneous interfacial region of the nanoparticles, polarity and electrostatic conditions differ markedly from the bulk solution.

Given that the anionic forms of quercetin absorb above 390 nm,^22^ the evident shoulder at 420 nm in the excitation spectrum of CTAB/H/Q in Fig. 4a and b can be attributed to the deprotonation of one or more OH groups. In order to check if differences in their emission properties with respect to those of the neutral quercetin are observed, fluorescence spectra were collected under excitation at 420 nm. No useful information was obtained, the spectra being identical to those obtained with excitation at 370 nm. This occurrence could indicate that the anionic forms of quercetin are not predominant when quercetin is loaded in the nanoparticles.

Due to the different fluorescence yields of these species, it is not possible to deduce information on their populations from these experimental data. Similarly, time-resolved fluorescence measurements reflect the superposition of lifetimes, with contributions dependent on population, quantum yields and emission wavelength.

The time-resolved fluorescence curves at *λ*_em_ = 610 nm (*λ*_ex_ = 370 nm) were fitted using a bi-exponential decay model for both free quercetin and for quercetin loaded CTAB/H nanoparticles (quercetin in the presence of CTAB alone exhibited too low fluorescence emission at 610 nm to collect data with good signal-to-noise ratio).

For free quercetin, there is a main fast component with a lifetime of about 0.15 ns and a smaller amplitude component with lifetime of about 1.3 ns (〈*τ*〉≈0.2 ns). No significant changes were observed in the presence of CTAB/H, which is consistent with the nearly unchanged emission band profile in the corresponding excitation spectra (Fig. 4b). Although it was not possible to isolate the fluorescence lifetimes of the two photo-tautomers due to the preponderant emission at 590 or 603 nm, the time-resolved fluorescence curves collected at 540 nm displayed an additional longer lifetime with a small amplitude, decreasing from 5 ns for free quercetin to 3.6 ns for quercetin loaded in CTAB/H nanoparticles. A similar lifetime value was measured for quercetin with CTAB alone (complete fit parameters are reported in Tables S3 and S4).

These data indicate that the emission of the excited tautomers of the neutral quercetin responds to the hydrophobic environment of the CTAB/H nanoparticles. For the tautomer emitting at lower wavelength, a shortening of the longer lifetime occurs (due to the low emission, no wavelength shift can be observed), whereas for that emitting at higher wavelength, a significant red shift in the steady-state fluorescence spectrum is observed (lifetimes are already too short for the used instrument to be sensitive to the effect of intermolecular interactions with the nanoparticle).

Thus, driven by hydrophobic interactions, quercetin is entrapped between the CTAB/H complexes of the nanoparticle, experiencing a very different environment compared to either the aqueous solvent or the CTAB molecules.

### Structural and morphological features

**Scheme 2:**
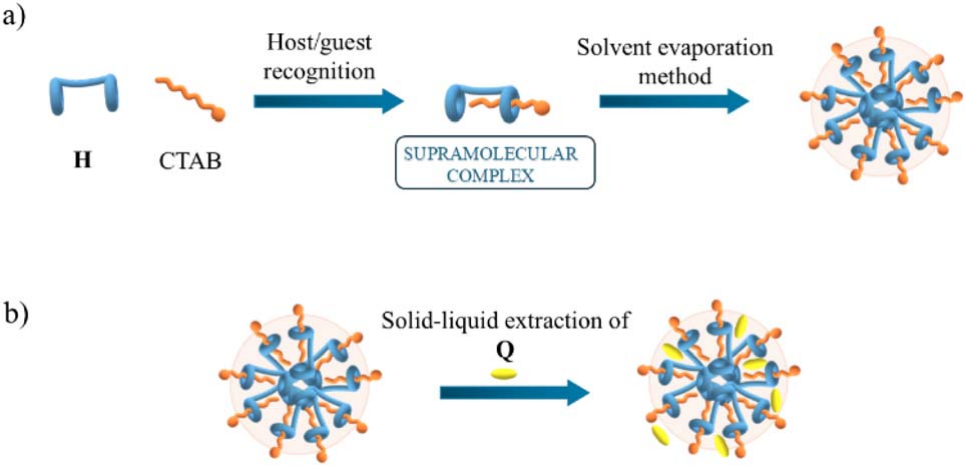
Sketch of the quercetin-loaded CTAB/H nanoparticles. Part (a) shows the formation of the CTAB/H supramolecular complex and part (b) depicts the hypothesis on the placement of the flavonol Q suggested by the experimental results.

To gain more insight into the interactions of quercetin upon binding with CTAB/H, structural information on the CTAB/H nanoparticles in solution is useful. Previous DLS experiments^12^ revealed that the average hydrodynamic radius of CTAB/H nanoparticles (approximately 40 nm) is initially determined by the thermodynamic equilibrium at the nanoscale between dispersed chloroform, in which H is dissolved, and the water from the CTAB solution (the formation of nanodomains of organic solvents in water is a common feature^34–36^). This is followed by the successive evaporation and sonication procedure (see the sketch in Scheme 2a). Therefore, the driving force that keeps the CTAB/H complexes together in the nanoparticle is essentially hydrophobic, with the charge of the polar CTAB head providing stable dispersion in water.

**Fig. 5:**
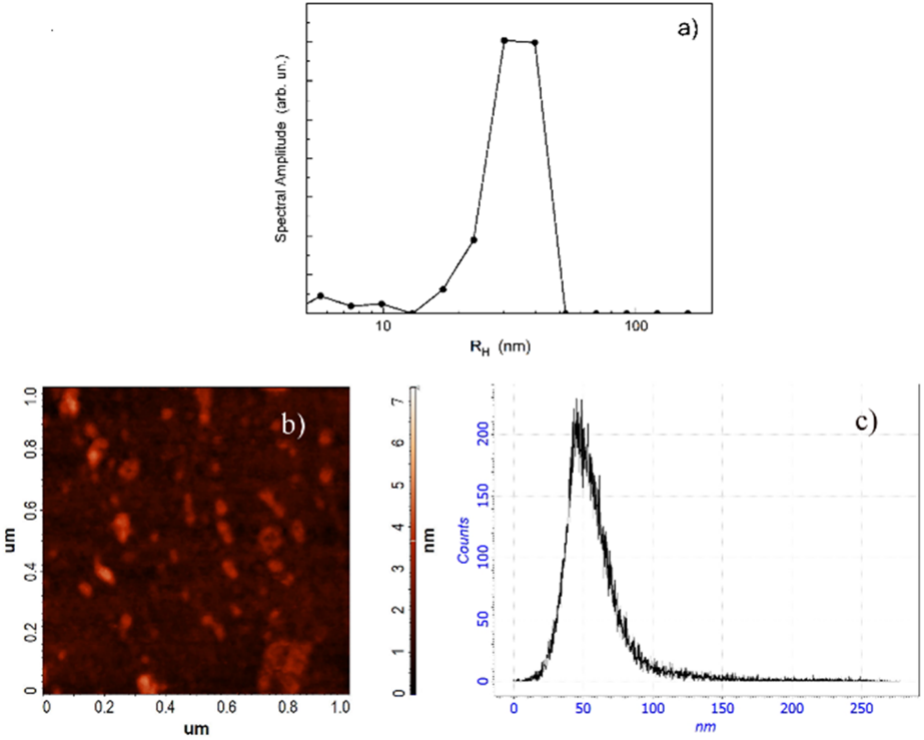
(a) Size distribution of CTAB/H/Q from DLS; (b) AFM morphology of the sample surface; (c) particle size distribution extracted from the AFM image (b), with an average particle radius of approximately 40 nm, in good agreement with DLS measurements.

DLS and AFM results indicate that the average nanoparticle size is not significantly affected by quercetin extraction. In fact, the size distribution from DLS (Fig. 5a) and the typical AFM map of CTAB/H/Q (Fig. 5b) with related particle size (Fig. 5c) agree in showing the presence of nanoparticles with radius around 40 nm. Larger particles are also clearly visible; these are likely attributable to a clustering process induced during solvent evaporation under UHV conditions.

CTAB/H nanoparticles do not possess an internal water pool (*i.e.* their structure is not vesicle-like). This was demonstrated by preparing the CTAB/H complex using a water solution of rhodamine B instead of pure water, followed by the addition of iodide (as potassium salt) as a quencher in the final solution. The quenching profile obtained upon titration indicates that rhodamine B was either in the bulk or in the proximity of the nanoparticle surface, but no fluorophore was protected from quenching in the inner part of the nanoparticles (Fig. S2-SI). Conversely, when CTAB/H/Q solution was subjected to iodide addition, no significant quenching effect was observed, suggesting that most quercetin cannot be reached by iodide ions, apart from the very small amount dissolved in water or at the nanoparticle surface (Fig. S2-SI). Furthermore, only when loaded in the CTAB/H complex, quercetin shows high stability against degradation over time (at least for one week), as confirmed by the invariance of the absorbance spectrum with time (see ref. 12 and Fig. S1c-SI).

As mentioned earlier, fluorescence spectra did not provide conclusive information about the role of the quercetin anionic form, which was further explored by measuring changes in the CTAB/H nanoparticles *ζ*-potential after quercetin extraction. As noted in ref. 12, CTAB/H nanoparticles have a positive surface charge (approximately +15 mV) due to the polar heads of the CTAB molecules emerging from the cavity of H. After quercetin extraction, the *ζ*-potential decreases to about +2 mV.

Taken together, the results indicate that most quercetin is loaded in the hydrophobic region of the nanoparticle, presumably in between the CTAB/H supramolecular complexes.

Moreover, the sub-micromolar traces of residual chloroform, remained entrapped in the nanoparticle, can provide further stabilization of the loaded quercetin. On the other hand, the decrease of the *ζ*-potential suggests that some quercetin molecules in the anionic form interact with the positive charges at the surface of the nanoparticles. The deduced structural hypothesis is sketched in Scheme 2b.

**Fig. 6:**
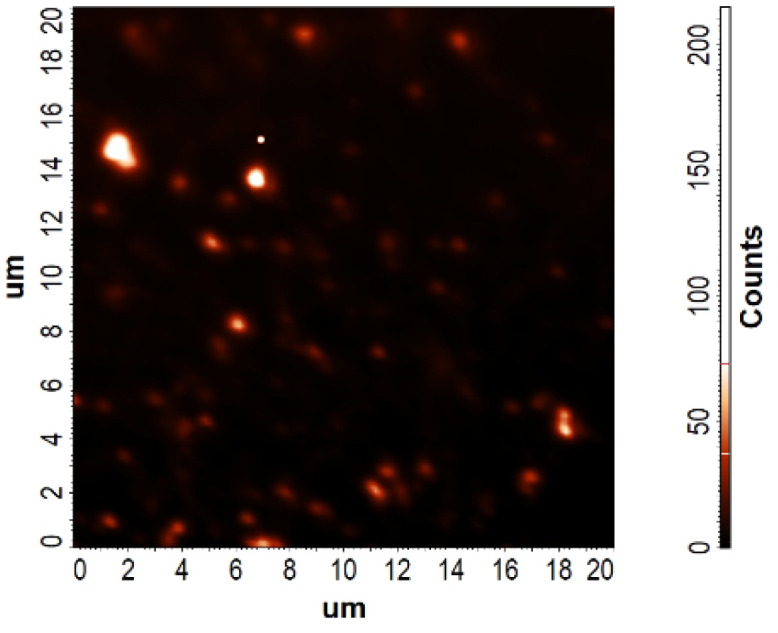
Fluorescence micrograph of quercetin-loaded nanoparticles. The field of view spans 20 × 20 µm. Due to the diffraction-limited resolution of the imaging system, the nanoparticles appear larger than their actual physical size.

To further investigate the location of quercetin, its fluorescence signal was collected from the surface of the drop-casted and dried sample. Bright quercetin-loaded nanoparticles were clearly observed (Fig. 6), although only the larger particles are distinctly visible due to the limitations imposed by the diffraction law.

## Conclusions

The self-assembly of CTAB/H supramolecular complexes leads to the formation of nanoparticles with a positively charged surface. The overall experimental evidence indicates that both neutral and mono-anionic forms of quercetin are inserted in the highly hydrophobic environment between the CTAB/H complexes in the nanoparticle. This arrangement protects quercetin from oxidation and degradation, explaining the previously reported success of the CTAB/H supramolecular complex in delivering and internalizing quercetin into cell cultures *in vitro*. A limited number of quercetin molecules in the anionic form are likely located at the nanoparticle surface, close to the CTAB polar heads, where they are not protected from degradation processes.

These results show how the CTAB/H supramolecular complexes organize into robust nanocarriers able to control the location and speciation of encapsulated molecules. This mechanistic insight offers useful guidelines for the rational design of pillararene-derived supramolecular delivery systems for poorly soluble bioactive compounds.

## Author contributions

Conceptualization, G. G. and V. V.; methodology, I. P., G. G., N. M. and V. V.; validation, I. P., A. N. and N. M.; formal analysis, S. P., I. P., N. M. and V. V.; investigation, M. M. (Marco Milone), M. M. (Martina Mazzaferro), S. P. and N. M.; writing—original draft preparation, V. V. and G. G.; writing—review and editing, A. N., I. P. and N. M.; visualization, M. M. (Marco Milone), M. M. (Martina Mazzaferro) and N. M.; project administration, I. P. and G. G.; funding acquisition, G. G. All authors have read and agreed to the published version of the manuscript.

## Conflicts of interest

There are no conflicts to declare.

## Supplementary Material

RA-016-D6RA02504H-s001

## Data Availability

Supporting data are reported in the supplementary information (SI). Supplementary information: effect of solutions aging on the absorption spectra (Fig. S1); tables with wavelength values and fluorescence intensity ratios of the main contributions in the fluorescence (Table S1) and excitation (Table S2) spectra; tables with the measured fluorescence lifetimes (Tables S3 and S4); graphics of the fluorescence quenching experiments (Fig. S2). See DOI: https://doi.org/10.1039/d6ra02504h.
