# Supplementary material for: Determining the microenvironment and protonation state of quercetin encapsulated in pillar[5]arene-based supramolecular nanocarriers
Source: RSC Adv. 2026 May 26;16(31):28435–41. doi: 10.1039/d6ra02504h (PMC13213575; doi:10.1039/d6ra02504h)
Supplement: RA-016-D6RA02504H-s001 [file RA-016-D6RA02504H-s001.pdf]

Supplementary Information (SI) for RSC Advances.
This journal is © The Royal Society of Chemistry 2026

Supplementary Information

DETERMINING THE MICROENVIRONMENT AND PROTONATION STATE OF QUERCETIN ENCAPSULATED IN PILLAR[5]ARENE-BASED SUPRAMOLECULAR NANOCARRIERS

Marco Milone,[a,†] Martina Mazzaferro,[a,†] Salvatore Patanè,[b] Anna Notti,[a] Ilenia Pisagatti,[a] Giuseppe Gattuso,[a] Norberto Micali [c] and Valentina Villari*[c]

[a]Dipartimento di Scienze Chimiche, Biologiche, Farmaceutiche ed Ambientali, Università degli Studi di Messina, Viale F. Stagno d'Alcontres 31, 98166 Messina, Italy.

[b]Dipartimento di Scienze Matematiche e Informatiche, Scienze Fisiche e Scienze della Terra, Università degli Studi di Messina, Viale F. Stagno d'Alcontres 31, 98166 Messina, Italy.

[c]CNR-IPCF Consiglio Nazionale delle Ricerche - Istituto per i Processi Chimico-Fisici, Viale F. Stagno d'Alcontres 37, 98158 Messina, Italy. E-mail: valentina.villari@cnr.it.

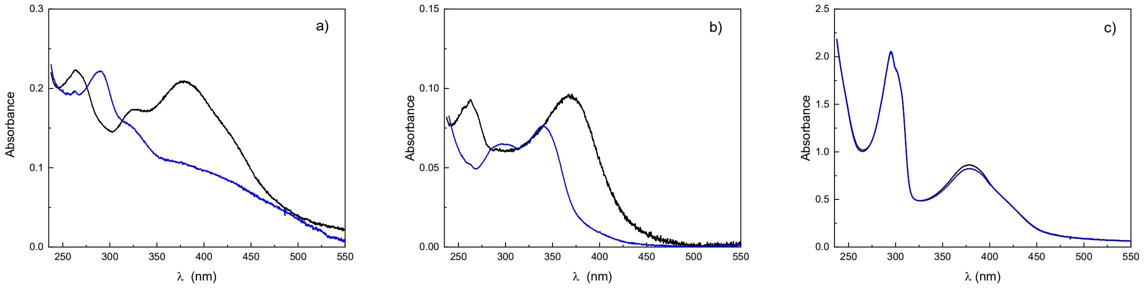

Figure S1. Effect of 1 week aging on quercetin: a) free; b) with CTAB; c) with CTAB/**H** nanoparticles (fresh solution: black curves, aged solutions: blue curves). The slight decrease observed at 378 nm for aged quercetin in the presence of CTAB/**H** can depend on some free quercetin molecules in the bulk (quercetin solubility in water is about 3.5 μg/mL[S1]) or at the nanoparticle surface.

Table S1. Wavelength values and fluorescence intensity ratios of the two main contributions in the fluorescence spectra (λ_{ex}=370 nm).

Sample	λ_1 (nm)	λ_2 (nm)	F_2/F_1
Q	542	593	9
CTAB/**H**/Q	-	603	-
CTAB/Q	545	593	3

S1 X. Ma, H. Su, Y. Liu, F. Chen and R. Xue, *RSC Adv.*, 2025, **15**, 9348–9358.

Table S2. Wavelength values and intensity ratios of the main contributions in the excitation spectra at two emission wavelengths.

	λ_{em}=540 nm					λ_{em}=610 nm				
Sample	λ_1 (nm)	λ_2 (nm)	λ_3 (nm)	I_1/I_2	I_1/I_3	λ_1 (nm)	λ_2 (nm)	λ_3 (nm)	I_1/I_2	I_1/I_3
Q	373	407	437	1	0.9	372	403	429	1.1	1.7
CTAB/**H**/Q	372	400	425	1.6	1.9	379	410	427	2.8	2.9
CTAB/Q	370	-	421	-	30	378	408	427	3.4	3.1

Table S3. Fluorescence lifetimes obtained at λ_{em}=610 nm with λ_{ex}=370 nm

Sample	A_1	τ_1 (±0.05 ns)	A_2	τ_2 (±0.05 ns)	$\langle\tau\rangle$ (ns)
Q	0.997	0.15	0.003	1.3	0.18
CTAB/**H**/Q	0.999	0.16	0.001	1.2	0.17
CTAB/Q*	-	-	-	-	-

*Fluorescence emission of CTAB/Q is too low for reliable determination of fluorescence lifetimes

Table S4. Fluorescence lifetimes obtained at λ_{em}=540 nm with λ_{ex}=370 nm

Sample	A_1	τ_1 (±0.05 ns)	A_2	τ_2 (±0.05 ns)	A_2	τ_2 (±0.1 ns)	$\langle\tau\rangle$ (ns)
Q	0.92	0.16	0.075	0.89	0.004	5.0	0.8
CTAB/**H**/Q	0.93	0.12	0.062	0.46	0.004	3.6	0.5
CTAB/Q	0.98	0.13	0.019	0.78	0.002	3.8	0.4

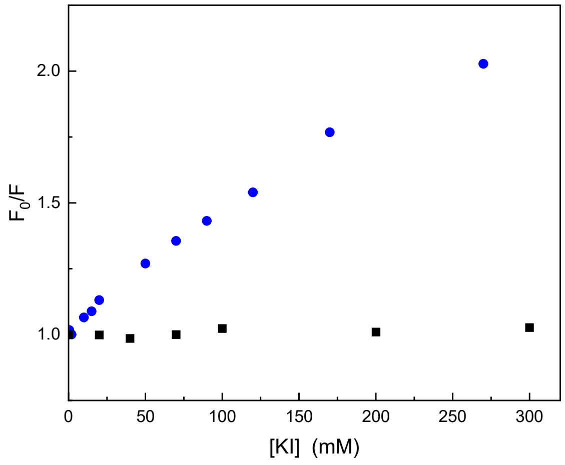

Figure S2. Fluorescence quenching experiment with the addition of potassium iodide (KI) for: CTAB/**H** nanoparticles prepared in a Rhodamine B aqueous solution (blue circles) and quercetin-loaded CTAB/**H** nanoparticles (black squares). See main text for more detail.
